# SNHG25 promotes colorectal cancer metastasis by regulating MMP2

**DOI:** 10.18632/aging.205060

**Published:** 2023-09-25

**Authors:** Tao Gong, Yu Li, Liang Feng, Qingyu Xu, Guoliang Dai, Min Li, Yi Wang, Shenlin Liu

**Affiliations:** 1 Oncology, Nanjing Hospital of Chinese Medicine, Nanjing Hospital of Chinese Medicine Affiliated to Nanjing University of Chinese Medicine, Nanjing 210023, China; 2 Oncology, Jiangsu Province Hospital of Chinese Medicine, Affiliated Hospital of Nanjing University of Chinese Medicine, Nanjing 210029, China; 3 School of Traditional Chinese Pharmacy, China Pharmaceutical University, Nanjing 211198, China; 4 Interventional Radiology Department, Jiangsu Cancer Hospital and Jiangsu Institute of Cancer Research and the Affiliated Cancer Hospital of Nanjing Medical University, Nanjing 210000, China; 5 Department of Clinical Pharmacology, Jiangsu Province Hospital of Chinese Medicine, Affiliated Hospital of Nanjing University of Chinese Medicine, Nanjing 210029, China; 6 Colorectal Surgery, Nanjing Hospital of Chinese Medicine, Nanjing Hospital of Chinese Medicine Affiliated to Nanjing University of Chinese Medicine, Nanjing 210023, China

**Keywords:** SNHG25, miR-296-3p, MMP2, colorectal cancer, lncRNA

## Abstract

LncRNA has been shown to play an important role in tumors, but the functions of most lncRNAs in colorectal cancer is not clear. By analyzing the transcriptome data of tumor tissues and adjacent tissues, we identified the lncRNA profiles that were abnormally expressed in colorectal cancer and selected the abnormally highly expressed lncRNA SNHG25 for further study. The functional assays showed that after knocking down SNHG25, the metastatic ability of colorectal cancer cells was significantly reduced. Western blot and immunofluorescence assays showed that inhibiting SNHG25 would affect the expression of Vimentin and E-Cadherin. In terms of mechanism, the results of RNA pull down assays, RNA immunoprecipitation (RIP) assays and dual luciferase reporter assays showed that SNHG25 could promote MMP2 expression by adsorbing miR-296-3p. In addition, chromatin immunoprecipitation (ChIP) assays and promoter luciferase reporter assays revealed that PAX5 could activate the transcription of SNHG25 in colorectal cancer cells. Our study proved that SNHG25 acts a pro-metastasis role in colorectal cancer, enriching the theory of the functions of lncRNA in colorectal cancer.

## INTRODUCTION

Colorectal cancer (CRC) stands as a prevalent malignancy affecting the digestive system. On a global scale, CRC witnessed approximately 1.9 million diagnoses and claimed the lives of around 925,000 individuals in the year 2020 [1]. Though ranked third in terms of incidence, it holds the second position in mortality rates. Notably, risk factors contributing to CRC include obesity, heightened consumption of animal-source foods, and adopting a more sedentary lifestyle [2, 3]. Early-stage CRC is mainly treated with surgery, and the prognosis is good. However, there is still a lack of effective treatment methods for advanced-stage CRC. At present, advanced-stage CRC is mainly treated with radiotherapy and chemotherapy, supplemented by targeted drug therapy, and the prognosis is generally poor [4, 5]. To further improve the prognosis of CRC patients, we need to clarify the molecular regulation mechanism of CRC and find novel effective diagnostic and therapeutic targets.

Only about less than 2% of the genes in the human genome are transcribed into mRNAs that encode proteins, and more than 80% of genes are transcribed into non-coding RNAs that do not have encode potential [6]. Among these non-coding RNAs, those with a length exceeding 200 nucleotides are classified as long non-coding RNAs (lncRNAs) [7]. Initially, these non-coding RNAs were considered mere “transcription noise” with no functional relevance. However, as research has deepened, it has become evident that non-coding RNAs play crucial roles in numerous physiological and pathological processes [8]. Within the context of tumors, lncRNAs have been found to influence various aspects such as tumor cell proliferation, apoptosis, migration, invasion, and drug resistance [9]. Although some lncRNAs have been linked to the onset and progression of CRC, the functional roles of most lncRNAs in colorectal cancer remain elusive [10, 11].

In our investigation, we unveiled a novel lncRNA known as SNHG25. Remarkably, SNHG25 exhibited a significant upregulation in CRC, and its heightened expression correlated with an unfavorable prognosis for CRC patients. Our research further demonstrated that SNHG25 plays a critical role in enhancing the metastatic capacity of CRC cells through the promotion of MMP2 expression. These findings strongly suggest that targeting SNHG25 could hold promise as a potential approach for the diagnosis and treatment of CRC.

## RESULTS

### SNHG25 is elevated in CRC, and high SNHG25 expression indicates a poor prognosis

**Figure 1:**
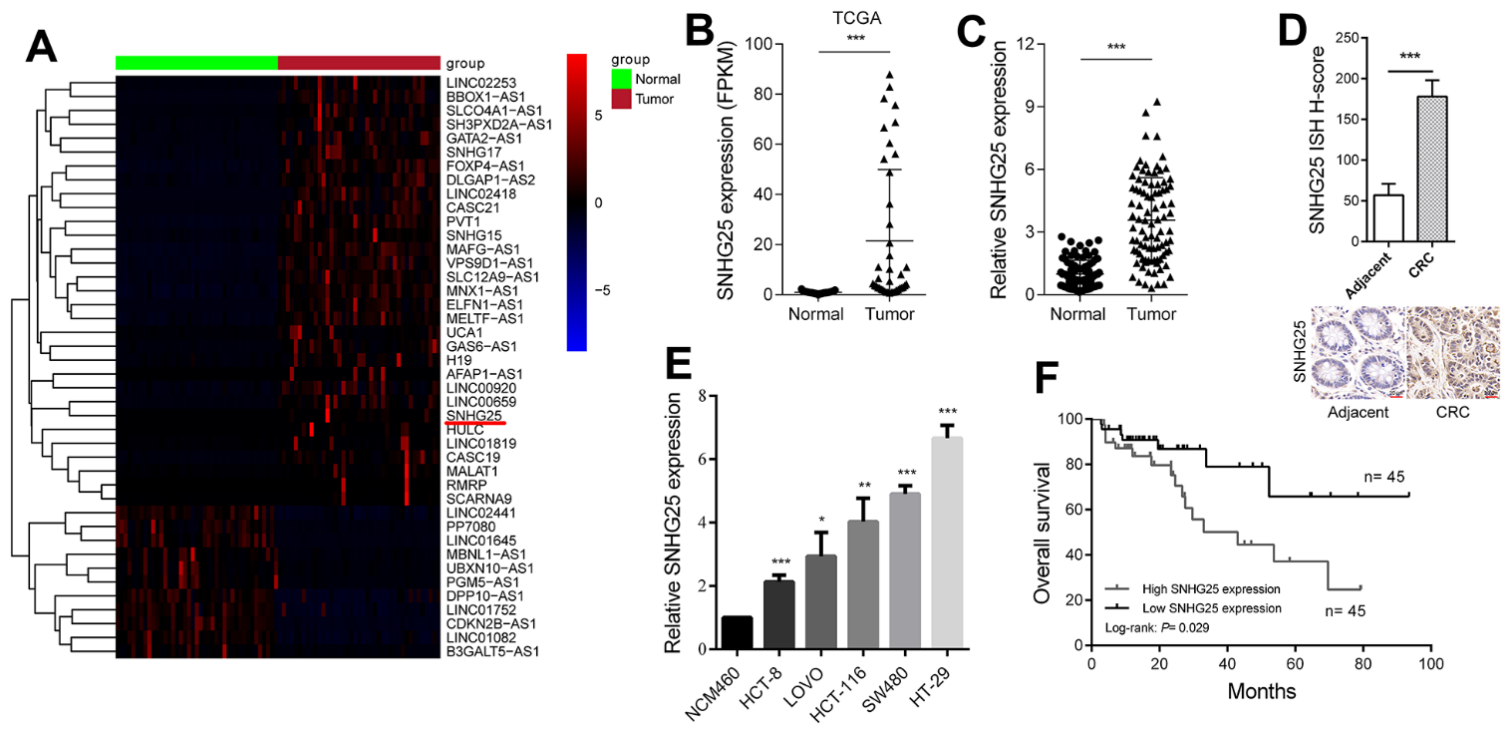
**SNHG25 is up-regulated in CRC and its expression level is related to prognosis.** (**A**) The heatmap shows the abnormal expression of lncRNAs in CRC in TCGA database. Red color indicates up-regulated in CRC, and blue color indicates down-regulated in CRC. SNHG25 is marked with a red underline. (**B**) Expression of SNHG25 in CRC generated from RNA sequencing data from TCGA database. (**C**) SNHG25 expression was detected by qRT-PCR in 90 pairs of CRC tissues and adjacent normal tissues. (**D**) Representative images of ISH assays for detecting SNHG25 in CRC tissues and corresponding normal tissues. (**E**) The expression of SNHG25 in NCM460, HCT-8, LOVO, HCT-116, SW480 and HT-29 cells was detected by qRT-PCR. (**F**) Kaplan-Meier survival curves and log-rank tests were used to compare the survival time between the high SNHG25 expression group and the low SNHG25 expression group. The top 50% of the samples were considered as high expression of SNHG25, and the bottom 50% samples considered as low SNHG25 expression. *P < 0.05, **P < 0.01 and ***P < 0.001.

Through an analysis of transcriptome sequencing data from both CRC tissues and adjacent normal tissues in the Cancer Genome Atlas (TCGA) database, we identified profiles of abnormally expressed lncRNAs in CRC (Figure 1A). Among these, we selected SNHG25 for further investigation. The sequencing data revealed a significant increase in SNHG25 expression in CRC tissues (Figure 1B). To validate these findings, we performed qRT-PCR on 90 pairs of CRC and adjacent tissues, confirming the upregulated expression of SNHG25 (Figure 1C). Additionally, ISH assays displayed higher SNHG25 expression in cancer tissue compared to adjacent normal tissue (Figure 1D). Moreover, we assessed SNHG25 expression in normal colorectal epithelial cells (NCM460) and five CRC cell lines (HCT-8, LOVO, HCT-116, SW620, and HT-29), clearly demonstrating that SNHG25 expression was significantly higher in CRC cells than in normal cells (Figure 1E). Notably, analysis of clinical data from CRC patients revealed that elevated SNHG25 expression correlated with tumor progression and a worse prognosis (Table 1 and Figure 1F).

**Table 1:** The clinical characteristics of 90 CRC patients.

**Characteristics**	**Number of cases**	**SNHG25 expression**		***p*-value^a^**
		**Low (n=45)**	**High (n=45)**	
**Age(year)**				
<60	36	16	20	0.389
≥60	54	29	25	
**Gender**				
Female	40	22	18	0.396
Male	50	23	27	
**Tumor invasion depth**				
T1-2	59	36	23	***0.004***
T3-4	31	9	22	
**Lymph node metastasis**				
N0	61	36	25	***0.013***
N1+N2	29	9	20	
**Distant metastasis**				
M0	74	41	33	***0.027***
M1+M2	16	4	12	
**TNM stage**				
I+II	57	34	23	***0.016***
III+III	33	11	22	

^a^Statistical significant results (in bold).

### The transcription factor PAX5 activates SNHG25 transcription in CRC

**Figure 2:**
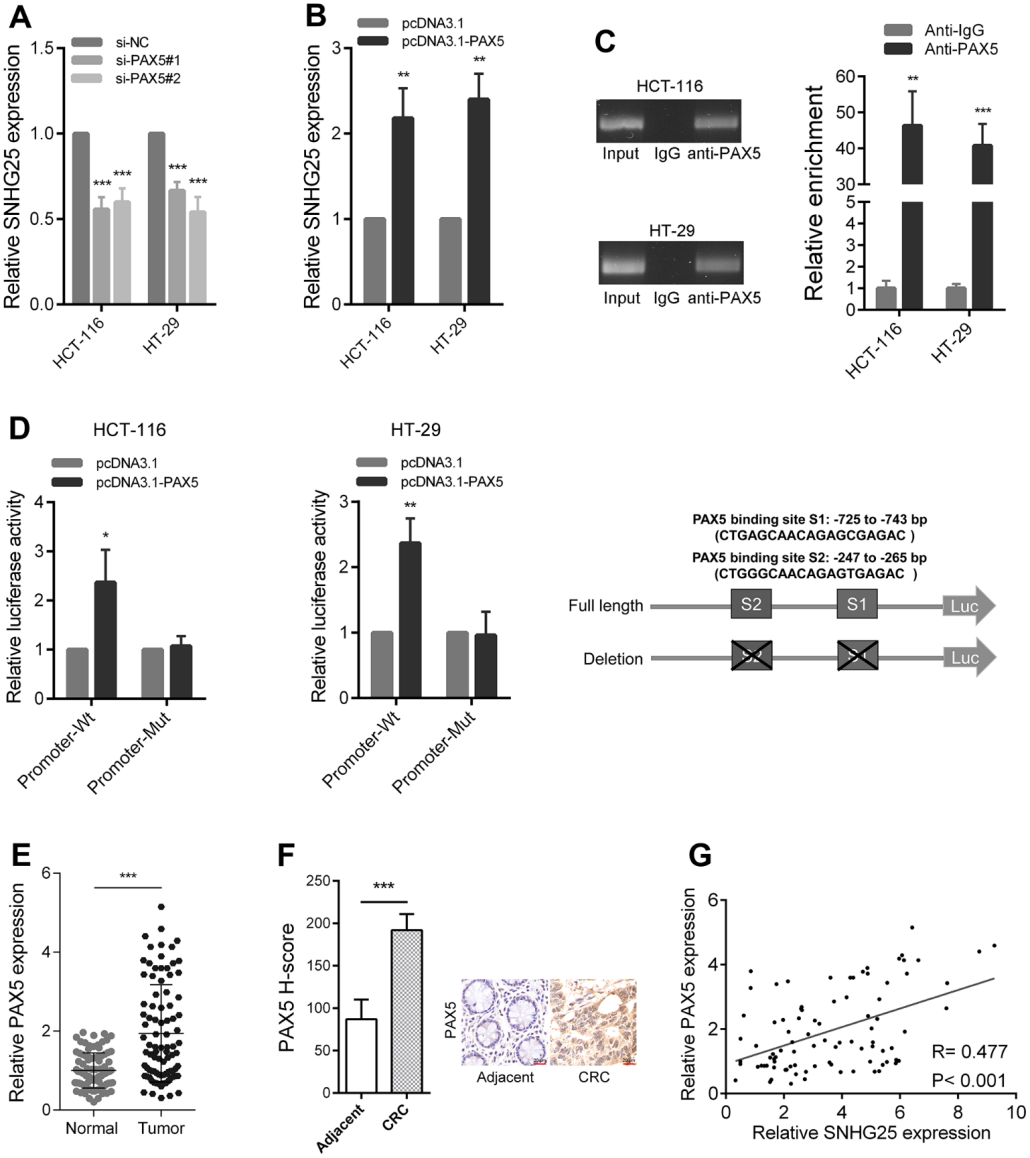
**PAX5 activates SNHG25 transcription in CRC.** (**A**) The expression of SNHG25 was detected by qRT-PCR in HCT-116 and HT-29 cells transfected with PAX5 siRNAs. (**B**) The expression of SNHG25 was detected by qRT-PCR in HCT-116 and HT-29 cells transfected with pcDNA3.1-PAX5 or pcDNA3.1. (**C**) ChIP-qPCR assays were performed to detect PAX5 occupancy at the SNHG25 promoter region. Genomic DNA input was 1%. (**D**) Luciferase reporter assays were used to determine the PAX5 binding sites on the SNHG25 promoter region. Schematic representation of constructs for reporter is in the right panel. (**E**) Relative expression of SNHG25 in 90 pairs of CRC tissues and corresponding normal tissues. (**F**) Representative images of PAX5 immunostaining of CRC tissues and corresponding normal tissues. (**G**) Correlation analysis between PAX5 and SNHG25 in 90 CRC tissues (R = 0.477, p < 0.001). *P < 0.05, **P < 0.01 and ***P < 0.001.

We used the JASPAR tool to find transcription factors that potentially bind to the SNHG25 promoter region. The results indicated that PAX5 obtained a highest score [12]. Upon silencing PAX5 in CRC cells, we observed a significant decrease in the expression level of SNHG25 (Figure 2A and Supplementary Figure 1A, 1B). Conversely, when PAX5 was overexpressed in CRC cells, there was a remarkable increase in SNHG25 expression (Figure 2B). Our ChIP assay results indicated direct binding of the PAX5 protein in CRC cells to the promoter region of SNHG25 (Figure 2C). To identify the specific binding site of PAX5 in the SNHG25 promoter, we designed two promoter luciferase reporter plasmids based on predicted sites. Co-transfection of the PAX5 overexpression plasmid (pcDNA3.1-PAX5) with the wild-type reporter plasmid (Promoter-Wt) resulted in a significant increase in luciferase activity. However, co-transfection with the mutant reporter plasmid (Promoter-Mut) lacking the binding site showed minimal change in luciferase activity (Figure 2D). Furthermore, we noted an upregulation of PAX5 expression in CRC (Figure 2E, 2F), and the expression level of PAX5 positively correlated with the expression level of SNHG25 in CRC tissues (Figure 2G).

### Knockdown SNHG25 reduces the metastatic ability of CRC cells

**Figure 3:**
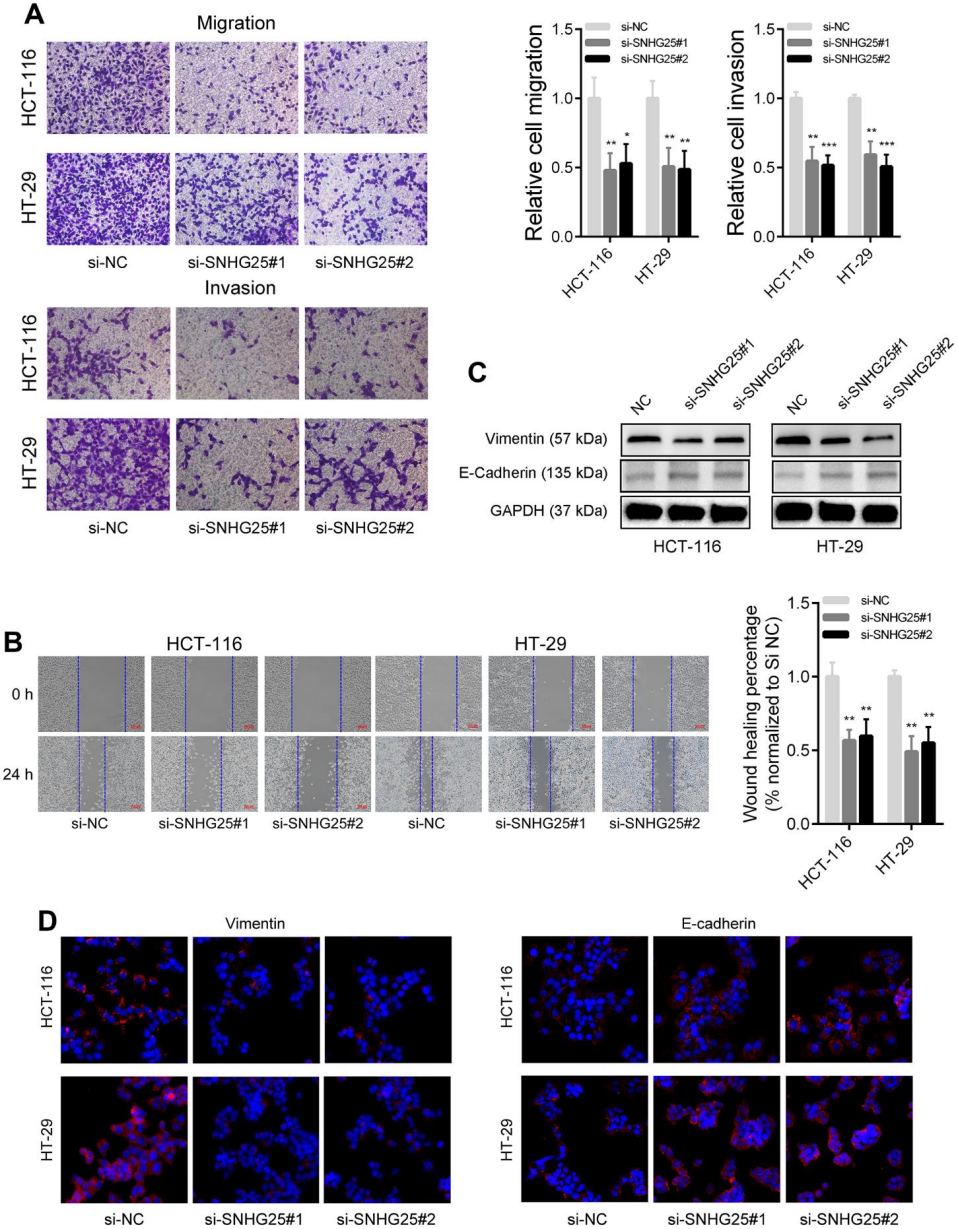
**SNHG25 regulates CRC cells metastasis.** (**A**) The migration and invasion abilities of HCT-116 and HT-29 cells were evaluated using transwell assays. (**B**) Representative images of wound healing assays performed using HCT-116 and HT-29 cells after SNHG25 silenced. (**C**) The expression of Vimentin and E-Cadherin were examined by western blot in SNHG25 knockdown HCT-116 and HT-29 cells. (**D**) The expression of Vimentin and E-Cadherin were detected by immunofluorescence after SNHG25 silencing. *P < 0.05, **P < 0.01 and ***P < 0.001.

The outcomes of the transwell migration and invasion assays demonstrated a significant reduction in the migration and invasion capabilities of CRC cells following the knockdown of SNHG25 (Figure 3A). Moreover, the wound-healing assays revealed a slower closure of scratch wounds in CRC cells transfected with SNHG25 siRNAs compared to the negative control cells (Figure 3B). Our western blot and immunofluorescence analyses further disclosed that inhibiting SNHG25 resulted in decreased Vimentin expression and increased E-Cadherin expression in CRC cells (Figure 3C, 3D).

### SNHG25 regulates MMP2 expression in CRC

**Figure 4:**
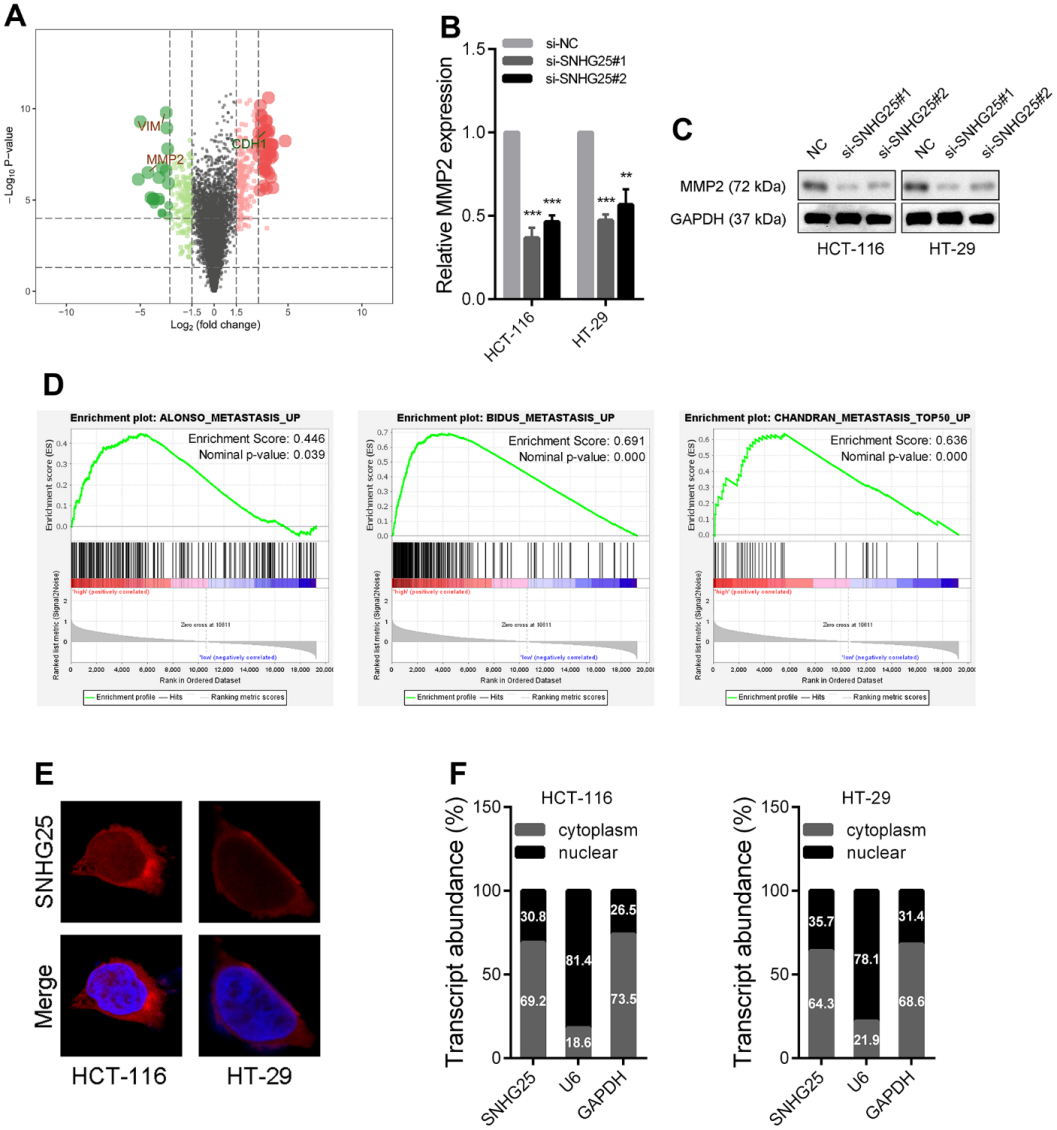
**The expression of MMP2 is regulated by SNHG25 in CRC.** (**A**) Volcano plot generate from the RNA-seq data showed the statistical significance (P value) and magnitude of change (fold change) of gene expression after SNHG25 knockdown. (**B**, **C**) The expression of MMP2 was detected by qRT-PCR or western blot in HCT-116 and HT-29 cells after SNHG25 knockdown. (**D**) GSEA results demonstrate the correlation between SNHG25 and gene sets related to metastasis (ALONSO_METASTASIS_EMT_UP, BIDUS_METASTASIS_UP and CHANDRAN_METASTASIS_TOP50_UP). (**E**) Representative FISH images demonstrated subcellar location of SNHG25 in HCT-116 and HT-29 cells (red). Nuclei were stained by DAPI (blue). (**F**) Relative SNHG25 expression in the nuclear and cytoplasm fractions of HCT-116 and HT-29 cells. Nuclear controls: U6, cytosolic controls: GAPDH. **P < 0.01 and ***P < 0.001.

The results of transcriptome sequencing after knockdown of SNHG25 in HCT-116 cells showed that the expression of several tumor metastasis-related genes changed significantly (MMP2, VIM and CDH1), among which the expression of MMP2 decreased most (Figure 4A and Supplementary Figure 2A, 2B). Consistent with the sequencing data, qRT-PCR and western blot results demonstrated that silencing SNHG25 obviously reduced the RNA and protein levels of MMP2 in CRC cells (Figure 4B, 4C). In addition, the results of Gene Set Enrichment Analysis (GSEA) showed that the expression of SNHG25 in CRC cells was related to some tumor metastasis gene sets (ALONSO_METASTASIS_EMT_UP, BIDUS_METASTASIS_UP and CHANDRAN_METASTASIS_TOP50_UP, Figure 4D).

We then explored how SNHG25 regulates MMP2 expression. The mechanism of lncRNA's function is partly dependent on its subcellular location. The results of FISH assays and qRT-PCR detection of nuclear and cytoplasmic components indicated that SNHG25 was mostly located in cytoplasm (Figure 4E, 4F).

### SNHG25 acts as molecular sponge to adsorb miR-296-3p in CRC

**Figure 5:**
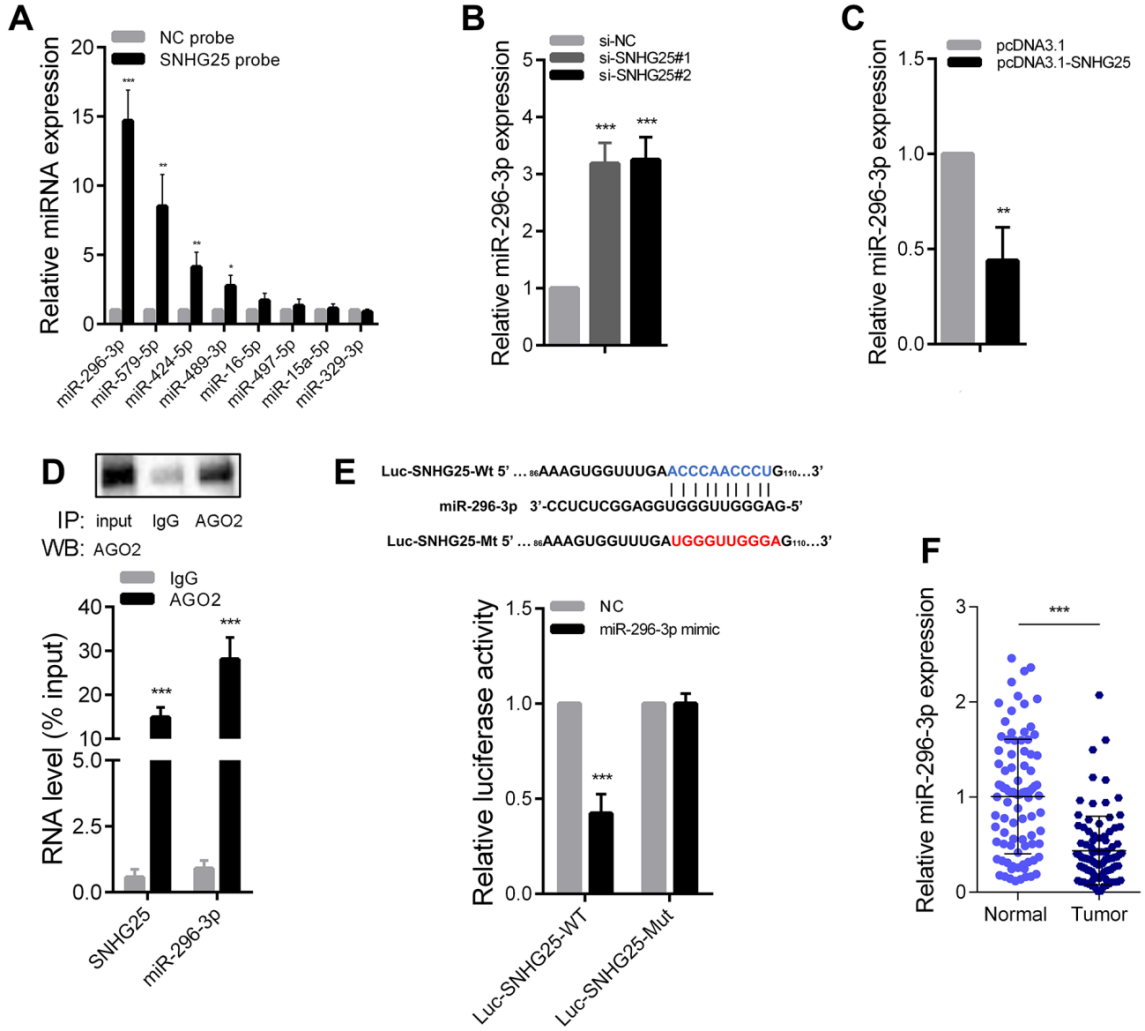
**SNHG25 acts as a sponge for miR-296-3p in CRC.** (**A**) Relative expression of microRNAs which potentially bind to SNHG25 were detected by qRT-PCR after RNA pull-down assays in HCT-116 cells with biotin labeled SNHG25. (**B**) MiR-296-3p expression was detected by qRT-PCR in HCT-116 cells transfected with SNHG25 siRNAs. (**C**) MiR-296-3p expression was detected by qRT-PCR in HCT-116 cells transfected with pcDNA3.1-SNHG25. (**D**) RIP assays were performed with antibodies against AGO2 or control IgG in HCT-116 cells. Immunoprecipitated RNA was analyzed by qRT-PCR. Total RNA input was 1%. (**E**) Dual luciferase reporter assays with wild type and mutant (binding sites for miR-296-3p were mutated) SNHG25 luciferase reporters. Up panel, sequence alignment of miR-296-3p and its potential binding sites in SNHG25. Predicted miR-296-3p target sequence (blue) in Luc-SNHG25-Wt and mutated nucleotides (red) in Luc-SNHG25-Mut. (**F**) MiR-296-3p expression was detected by qRT-PCR in 90 pairs of CRC tissues and adjacent normal tissues. *P < 0.05, **P < 0.01 and ***P < 0.001.

Previous research has established that cytoplasmic lncRNAs can function as “molecular sponges”. To identify potential microRNAs that interact with SNHG25, we conducted an RNA pull-down experiment using biotin-labeled SNHG25 in HCT-116 cells. Subsequently, qRT-PCR was utilized to assess the abundance of microRNAs bound to SNHG25, revealing that miR-296-3p exhibited the highest binding affinity (Figure 5A). Additionally, silencing SNHG25 in HCT-116 cells led to a significant increase in miR-296-3p expression, while overexpression of SNHG25 resulted in a notable decrease in miR-296-3p levels (Figure 5B, 5C). Furthermore, the RIP experiment using the AGO2 antibody provided evidence that SNHG25 and miR-296-3p could bind to the AGO2 protein concurrently (Figure 5D). To determine the specific binding sites of miR-296-3p within SNHG25, we designed dual luciferase reporter constructs based on the predicted binding sites from bioinformatics analysis. As depicted in Figure 5E, miR-296-3p overexpression substantially reduced the luciferase activity produced by the wild-type reporter plasmid (Luc-SNHG25-Wt), while leaving the luciferase activity of the mutant reporter plasmid (Luc-SNHG25-Mut) unaffected. Additionally, we conducted qRT-PCR to detect miR-296-3p expression in 90 pairs of CRC and adjacent tissues, confirming that miR-296-3p expression was significantly down-regulated in CRC tissues compared to normal tissues (Figure 5F).

### SNHG25 enhances the expression of MMP2 by adsorbing miR-296-3p in CRC

**Figure 6:**
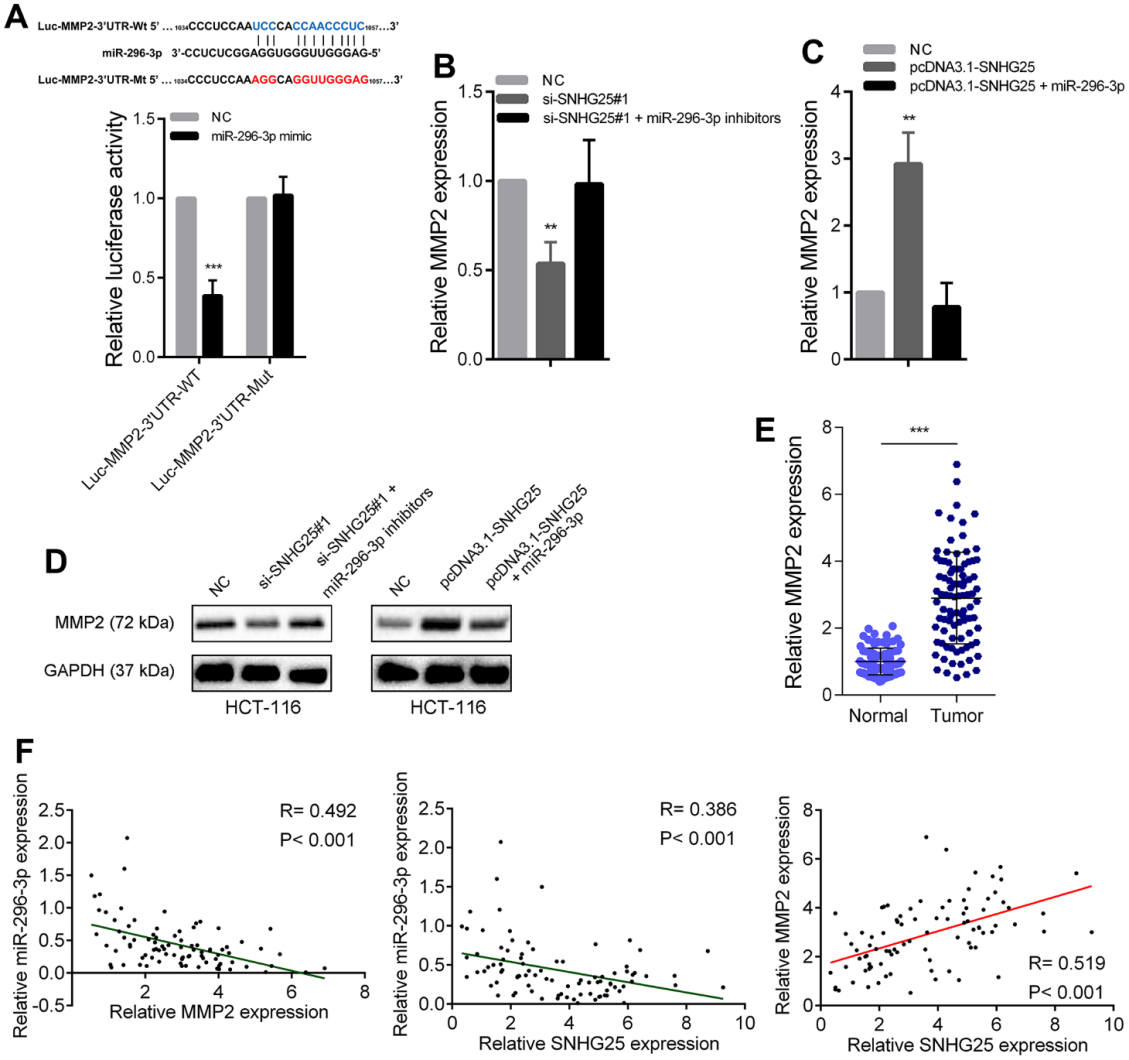
**SNHG25 promoted MMP2 expression by adsorbing miR-296-3p.** (**A**) Dual luciferase reporter assays with wild type or mutant MMP2-3’UTR luciferase reporters after transfection of miR-296-3p mimics. Upper panel, sequence alignment of miR-296-3p and its potential binding sites in MMP2 3’UTR. Predicted miR-296-3p target sequence (blue) in Luc-MMP2-3’UTR-Wt and mutated nucleotides (red) in Luc-MMP2-3’UTR-Mut. (**B**) MMP2 expression was detected by qRT-PCR after transfection of SNHG25 siRNAs or co-transfection of SNHG25 siRNAs and miR-296-3p inhibitors in HCT-116 cells. (**C**) MMP2 expression was detected by qRT-PCR after transfection of pcDNA3.1-SNHG25 or co-transfection of pcDNA3.1-SNHG25 and miR-296-3p mimics in HCT-116 cells. (**D**) MMP2 expression was detected by western blot in HCT-116 cells with indicated treatments. (**E**) qRT-PCR analysis of MMP2 expression in 90 pairs of CRC and corresponding adjacent normal tissues. (**F**) Correlation analysis between SNHG25, MMP2 and miR-296-3p in 90 paired CRC samples (miR-296-3p vs. SNHG25, R = 0.386, p < 0.001; miR-296-3p vs. MMP2, R = 0.492, p < 0.001; MMP2 vs. SNHG25, R = 0.519, p < 0.001). **P < 0.01 and ***P < 0.001.

Through the prediction of bioinformatics tools, we found that MMP2 is also a potential direct target of miR-296-3p. We designed dual luciferase reporters according to the binding sites of miR-296-3p in MMP2’ 3’UTR. As shown in Figure 6A, overexpression of miR-296-3p could cause a significant decrease in luciferase activity, but when the binding sites were mutated, the overexpression of miR-296-3p would not cause a significant change in luciferase activity. In addition, MMP2 expression was significantly reduced after miR-296-3p overexpression in CRC cells and significantly increased after miR-296-3p inhibition (Supplementary Figure 2C, 2D). We further explored whether SNHG25 regulated MMP2 expression by adsorbing miR-296-3p. After knocking down SNHG25 in HCT-116 cells, the expression level of MMP2 decreased significantly. Knockdown of SNHG25 while inhibiting miR-296-3p, the reduction of MMP2 expression was rescued (Figure 6B). Conversely, when SNHG25 was overexpressed in HCT-116 cells, the expression level of MMP2 was increased, and when SNHG25 and miR-296-3p were co-overexpressed, the elevation of MMP2 was lost (Figure 6C). The change trend of MMP2 in protein level is consistent with RNA level (Figure 6D). In addition, qRT-PCR results showed that MMP2 expression was significantly up regulated in CRC (Figure 6E). Correlation analysis showed that in CRC tissues, the expression of SNHG25 and MMP2 were negatively correlated with the expression of miR-296-3p and the expression of SNHG25 and MMP2 was positively correlated (Figure 6F).

### The role of SNHG25 in promoting CRC metastasis is partly dependent on the regulation of MMP2

**Figure 7:**
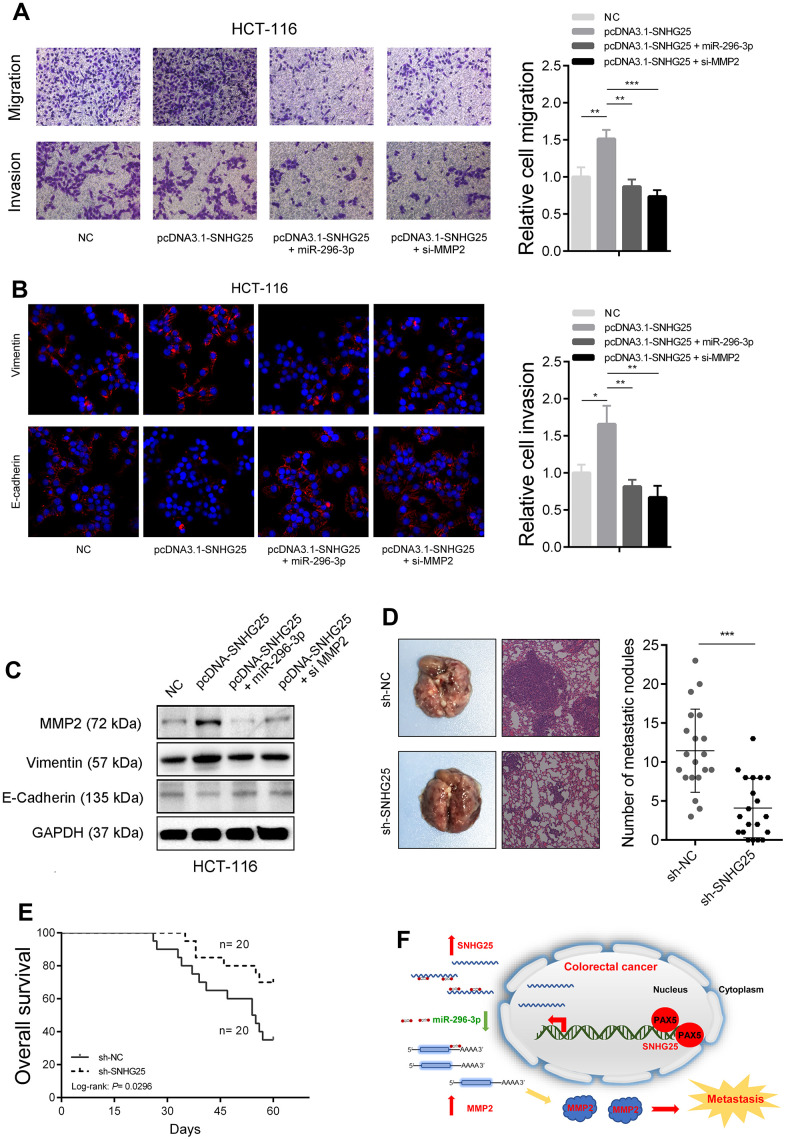
**SNHG25 promotes CRC metastasis by regulating MMP2 expression.** (**A**) The migration and invasion abilities of HCT-116 cells were evaluated by transwell assays after indicated treatments. (**B**) Vimentin and E-Cadherin expression was detected by immunofluorescence in HCT-116 cells with indicated treatments. (**C**) MMP2, Vimentin and E-cadherin expression was detected by western blot in HCT-116 cells with indicated treatments. (**D**) Left panel, representative images of lungs from mice after tail vein injections with stably transfected sh-SNHG25 and sh-NC HCT-116 cells. Right panel, quantitative analysis of metastasis foci in corresponding groups. (**E**) Survival analysis of mice after tail vein injections with stably transfected sh-SNHG25 and sh-NC HCT-116 cells. (**F**) Schematic of the proposed mechanism of SNHG25 in CRC. *P < 0.05, **P < 0.01 and ***P < 0.001.

Subsequently, we investigated whether SNHG25's promotion of CRC cell metastatic capability was contingent on its regulation of MMP2. The transwell assays revealed that SNHG25 overexpression notably enhanced the migration and invasion abilities of HCT-116 cells. However, when miR-296-3p was simultaneously overexpressed or MMP2 was silenced, the augmented migration and invasion abilities induced by SNHG25 overexpression were mitigated (Figure 7A and Supplementary Figure 2E). Furthermore, the results of immunofluorescence and western blot analyses exhibited that SNHG25 overexpression led to an increase in Vimentin expression and a decrease in E-Cadherin expression. Nevertheless, when miR-296-3p was concurrently overexpressed or MMP2 was inhibited, these changes induced by SNHG25 overexpression were attenuated (Figure 7B, 7C). Finally, we further substantiated the impact of SNHG25 on the metastatic potential of CRC cells through *in vivo* assays. The results demonstrated that the number of lung nodules formed from SNHG25 knockdown HCT-116 cells was significantly reduced compared to the control group. Additionally, the survival time of mice in the SNHG25 knockdown group was significantly prolonged compared to the control group (Figure 7D, 7E and Supplementary Figure 2F).

## DISCUSSION

An increasing number of studies have focused on roles of lncRNA in CRC. Liu et al. reported that lncRNA MIR100HG affected m6A-dependent stabilization of TCF7L2 mRNA in CRC by interacting with hnRNPA2B1 [13]. Chen et al. found SP1-mediated lncRNA ZFAS1 could promote CRC metastasis by up-regulating VEGFA [14]. Wu et al. demonstrated that lncRNA SLCO4A1-AS1 facilitates CRC stem cell self-renewal through increasing SLCO4A1 expression [15]. In this study, we conducted an analysis of the sequencing data from CRC tissues in the TCGA database to identify lncRNAs with abnormal expression patterns. Among these lncRNAs, SNHG25 was chosen for in-depth investigation. Our findings revealed a significant upregulation of SNHG25 expression in both CRC tissues and cell lines, and this elevated expression correlated with a poor prognosis in CRC patients. Furthermore, we provided evidence that PAX5 plays a role in activating the transcription of SNHG25 in CRC. Importantly, through *in vivo* and *in vitro* experiments, we demonstrated that SNHG25 functions as a promoter of CRC cell metastasis. In terms of molecular mechanism, we found that SNHG25 was mainly located in the cytoplasm, and it could adsorb miR-296-3p to increase the expression of MMP2 (Figure 7F). Our data reveals that SNHG25 plays an oncogene role in CRC and suggests that it can be a potential diagnosis and treatment target for CRC.

SNHG25 is located on human chromosome 17q23. At present, there are few studies on the function of SNHG25. Wu et al. proved that SNHG25 facilitated Glioma progression by activating MAPK pathway [16]. Zeng et al. found SNHG25 recruited dyskerin pseudouridine synthase 1 to stabilize HDAC1 in neuroblastoma cells [17]. We firstly report the role of SNHG25 in CRC and elucidate the underlying molecular mechanism. Previous studies have indicated that the expression of many lncRNAs in tumors is regulated by transcription factors. In this study, we found that the transcription factor PAX5 could activate the transcription of SNHG25 in CRC cells. PAX5 is a member of the paired box (PAX) family of transcription factors. It has many different functions, including in neural development and spermatogenesis. It was also found to play a vital role in neoplastic transformation [18, 19]. Wang et al. found that the expression of PAX5 was significantly increased in CRC and could facilitate CRC cell growth. This conclusion is consistent with our results. However, they found that PAX5 could promote the expression of UASR1, which was different from our target [20]. Many lncRNAs located in the cytoplasm have been reported to act as molecular sponges to adsorb microRNAs [21, 22]. In this study, we revealed that SNHG25 could adsorb miR-296-3p and promote the expression of MMP2. MiR-296-3p has been demonstrated to play a tumor suppressor role in a variety of tumors [23–25]. In CRC, miR-296-3p was found to inhibit tumor cell proliferation by suppressing GRINA expression [26]. Besides, Li et al. found that miR-296-3p weakened cancer stem cell-like properties through targeting TGIF1 and regulated by HDAC3, which formed a feedback loop in CRC [27]. Interestingly, Wang et al. found that miR-296-3p could both regulate MMP2 and MMP9 in choroidal malignant melanoma [28]. Our results also proved miR-296-3p could inhibit MMP2 expression independently.

MMP2 is a widely reported pro-metastasis gene [29–31]. Jiang et al. revealed that β3GnT8 promoted CRC invasiveness and metastasis by activating MMP2/Galectin3 axis [32]. In addition, Lu et al. suggested that Twist1/2 promoted CRC metastasis through activating MMP2 transcription [33]. Our data revealed another signaling pathway which could regulate MMP2 expression in CRC.

In conclusion, our research emphasizes the oncogenic role of SNHG25 in promoting the metastasis of CRC cells. Through in-depth mechanistic analysis, we unveil a novel regulatory network involving SNHG25, miR-296-3p, and MMP2, which contributes to the progression of CRC.

## MATERIALS AND METHODS

### Tissue samples and clinical data collection

Tissue samples were procured from 90 patients with colorectal cancer (CRC) undergoing surgery at the Nanjing Hospital of Chinese Medicine between 2014 and 2020. Paired noncancerous tissues were also collected from the same patients. Prior to the operation, none of the patients had received any anticancer treatment. The collected samples were rapidly frozen in liquid nitrogen and subsequently stored at -80° C until they underwent RNA analysis.

### Cell culture

The human colorectal cancer cell lines HCT-8, HCT-116, and HT-29 were acquired from ATCC, USA, while the human colorectal cancer cell lines LOVO and SW480 were obtained from Beyotime, China. Additionally, the normal colorectal epithelial cell line NCM460 was purchased from Procell, China. To ensure authenticity, all cell lines underwent validation through STR analysis. HCT-8, HT-29, LOVO, and SW480 cells were cultured in DMEM medium supplemented with 10% fetal bovine serum and antibiotics (penicillin 100 U/mL, streptomycin 0.1 mg/mL). On the other hand, NCM460 and HCT-116 cells were cultured in RPMI 1640 medium containing 10% FBS and antibiotics.

### Transfection

The small interfering RNA (siRNA) targeting SNHG25, PAX5, and their corresponding negative controls, along with short hairpin RNAs (sh-RNAs) for SNHG25, pcDNA3.1-SNHG25, and pcDNA3.1-PAX5 were obtained from GenePharma (China). Additionally, miR-142-3p mimics and inhibitors were purchased from RiboBio (China). Transfection was carried out using Lipofectamine 3000 (Invitrogen, USA).

### Quantitative real-time PCR

Tissue or cell total RNA was isolated using the Trizol reagent (Ambion, USA) following the recommended protocol. Subsequently, cDNA synthesis was carried out using the PrimeScriptTM RT Master Mix Kit (TaKaRa, Japan). For quantitative real-time PCR (qRT-PCR), the SYBR Green Premix Ex Taq (TaKaRa, Japan) was utilized, and the analysis was conducted on an ABI 7500 system. The primer sequences used were as follows: for SNHG25, GCAGGTTCCGGGAGGTCA (forward) and CAAACCACTTTATTGACGGGAA (reverse); for MMP2, AGCGAGTGGATGCCGCCTTTAA (forward) and CATTCCAGGCATCTGCGATGAG (reverse); for PAX5, CTTGCTCATCAAGGTGTCAGGC (forward) and TGGCGACCTTTGGTTTGGATCC (reverse); for GAPDH, GGAGCCAAAAGGGTCATCACTC (forward) GAGGGGCCATCCACAGTCTTCT (reverse).

### Western blot

In summary, the protein extraction, quantification, and separation were performed using 10% SDS-PAGE, followed by the transfer to PVDF membranes (Millipore, USA). The membranes were then incubated at room temperature for 1 hour with 5% skim milk and primary antibodies against Vimentin (#5741, Cell Signaling Technology, USA), E-Cadherin (#3195, Cell Signaling Technology, USA), MMP2 (#40994, Cell Signaling Technology, USA), and Argonaute-2 (#ab186733, Abcam, USA). Subsequently, the membranes were exposed to HRP-labeled secondary antibodies and visualized using chemiluminescence. As a control, Anti-GAPDH (#2118, Cell Signaling Technology, USA) was utilized.

### Transwell assay

The cell migration and invasion capabilities were assessed using 24-well transwell units equipped with polycarbonate filters (Corning Costar, USA). Following transfection, 5 × 10^4^ CRC cells were suspended in 100 μl of FBS-free medium and placed in the upper chambers. In the lower compartment, 700 μl of medium containing 20% FBS was added. After a 48-hour incubation period, cells adhering to the lower surface of the chamber were fixed using 4% ethanol for 30 minutes and subsequently stained with 0.1% crystal violet for 30 minutes. Cell counting was performed in five randomly chosen fields under a microscope. For the invasion assay, chambers coated with matrigel were utilized.

### Chromatin immunoprecipitation assay

The chromatin immunoprecipitation (ChIP) assay was conducted employing the Magna ChIP Kit (Merk-Millipore, USA). In summary, cells were fixed using a 1% formaldehyde solution for 20 minutes, and subsequently, glycine was added to achieve a final concentration of 125 mM while shaking for 5 minutes. Then the cross-linked chromatin DNA was cut into 200bp to 1000bp fragments, and immunoprecipitated with anti-PAX5 antibody (#ab109443, Abcam, USA). ChIP primer for the SNHG25 promoter: AATCAAAGCCAACAGCCAAC (forward) and ATGAACAGCCTACTGTAGAA (reverse).

### Luciferase reporter assays

To construct the luciferase reporter plasmids, we separately sub-cloned the wild-type fragments containing miR-142-3p binding sites (SNHG25-Wt and MMP2-3’UTR-Wt) and their corresponding mutant fragments (SNHG25-Mut and MMP2-3’UTR-Mut) downstream of the pmirGLO Vector (Promega, USA). Moreover, we created pGL3/Basic vector (Promega, USA) containing the SNHG25 promoter with PAX5 binding sites (promoter-Wt) or with mutations (promoter-Mut). Following a 48-hour transfection period, we employed the Dual-Luciferase Reporter Assay System (Promega, USA) to measure the luciferase activity.

### RNA fluorescence *in situ* hybridization

Cy3-labeled probes specific to SNHG25 were custom-designed and synthesized by RiboBio (China). For the Fluorescent *In Situ* Hybridization (FISH) assays, the Fluorescent *In Situ* Hybridization Kit (RiboBio, China) was employed, following the provided protocol.

### RNA pull-down assay

The RNA pull-down assay was performed using the biotinylated RNA pull-down Kit (BersinBio, China). Briefly, a total of 10^7^ HCT-116 cells were cross-linked by ultraviolet irradiation and lysed with 1 ml lysis buffer. 3 μg biotinylated probes of SNHG25 were hybridized with lysis and then incubated with streptavidin-coated magnetic beads. Non-specifically bound RNAs were washed, and bound miRNAs was detected by qRT-PCR.

### Animal experiments

For the tail vein injection lung metastasis model, 1.5 × 10^6^ SNHG25 stably knockdown or control HCT-116 cells were tail-vein injected into five-week-old male BALB/c nude mice. When the mouse dies, record the survival time, and surgically removed the lung and stored in formalin. All mice were killed after two months. The lung tissues were stained by H&E, and the metastatic nodules were counted. All animal experiments were performed in accordance with the NIH Guidelines for the Care and Use of Laboratory Animals and approved by the Ethics Committee of the Nanjing Hospital of Chinese Medicine.

### Statistical analysis

The statistical analysis was carried out using either SPSS 20.0 (IBM, USA) or Prism 5 (GraphPad Software, USA). All experimental data were expressed as the mean ± standard deviation (S.D.) and derived from a minimum of three independent experiments. To compare two independent groups, two-tailed Student's t-test or one-way ANOVA was employed. For survival curve analysis, the log-rank test was applied. Pearson's correlation coefficients were utilized to analyze correlations between variables. A *p*-value of less than 0.05 was considered statistically significant.

## Supplementary Material

Supplementary Figures
