# Supplementary material for: SNHG25 promotes colorectal cancer metastasis by regulating MMP2
Source: Aging (Albany NY). 2023 Sep 25;15(19):10105–16. doi: 10.18632/aging.205060 (PMC10599716; doi:10.18632/aging.205060)
Supplement: Supplementary Figures [file aging-15-205060-s001.pdf]

SUPPLEMENTARY FIGURES

Supplementary Figures

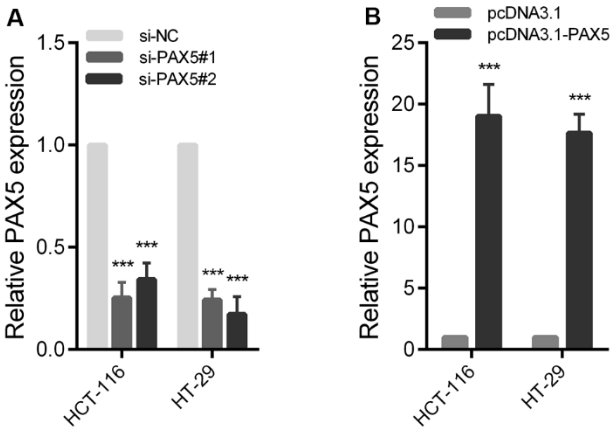

Supplementary Figure 1. (**A**) PAX5 expression was detected by qRT-PCR in HCT-116 cells transfected with PAX5 siRNAs. (**B**) PAX5 expression was detected by qRT-PCR in HCT-116 cells transfected with pcDNA3.1-PAX5. ***P < 0.001.

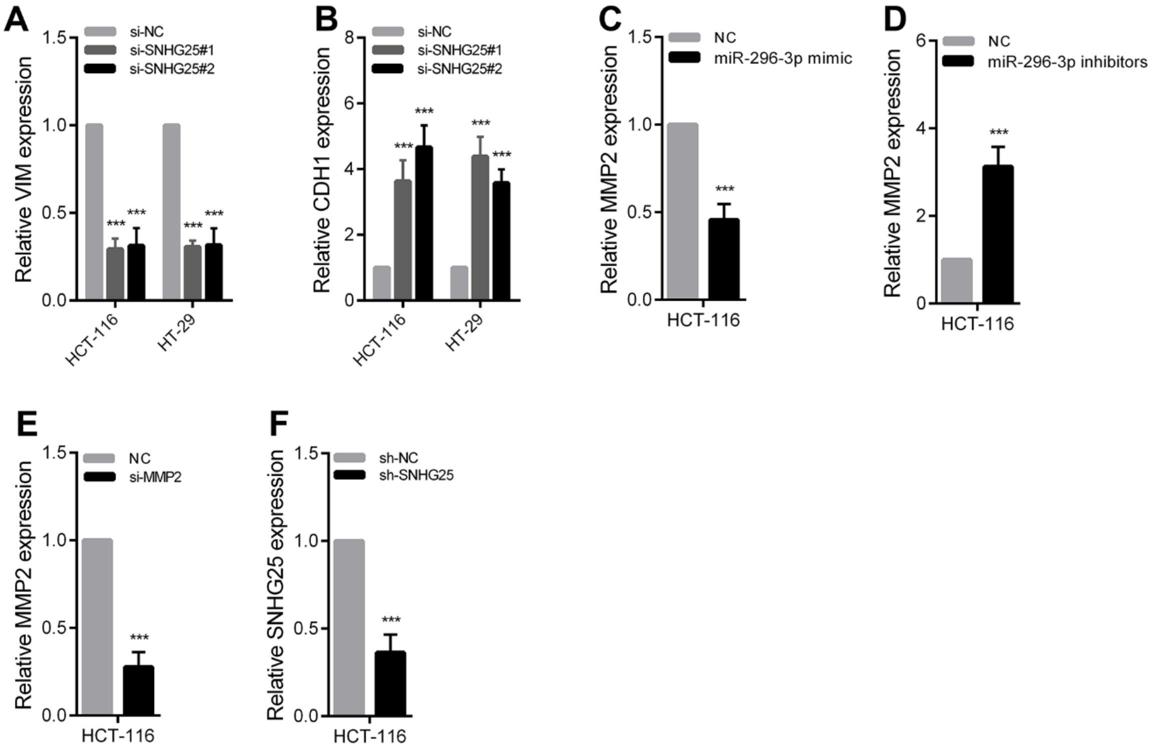

Supplementary Figure 2. (**A**, **B**) VIM and CDH1 expression was detected by qRT-PCR in HCT-116 cells after SNHG25 knockdown. (**C**) MMP2 expression was detected by qRT-PCR after transfection of miR-296-3p mimics in HCT-116 cells. (**D**) MMP2 expression was detected by qRT-PCR after transfection of miR-296-3p inhibitors in HCT-116 cells. (**E**) MMP2 expression was detected by qRT-PCR in HCT-116 cells after transfection with MMP2 siRNAs. (**F**) SNHG25 expression was detected by qRT-PCR in tissues stably transfected sh-SNHG25 and sh-NC plasmid. ***P < 0.001.
